# Cognitive Architecture with Evolutionary Dynamics Solves Insight Problem

**DOI:** 10.3389/fpsyg.2017.00427

**Published:** 2017-03-29

**Authors:** Anna Fedor, István Zachar, András Szilágyi, Michael Öllinger, Harold P. de Vladar, Eörs Szathmáry

**Affiliations:** ^1^Parmenides Center for the Study of Thinking, Parmenides FoundationPullach am Isartal, Germany; ^2^MTA-ELTE Theoretical Biology and Evolutionary Ecology Research GroupBudapest, Hungary; ^3^Institute of Advanced Studies Kőszeg (iASK)Kőszeg, Hungary; ^4^Department of Plant Systematics, Ecology and Theoretical Biology, Eötvös Loránd University (ELTE)Budapest, Hungary; ^5^Center for the Conceptual Foundations of Science, Parmenides FoundationPullach am Isartal, Germany

**Keywords:** insight, Darwinian Neurodynamics, attractor networks, four-tree problem, evolutionary search

## Abstract

In this paper, we show that a neurally implemented a cognitive architecture with evolutionary dynamics can solve the four-tree problem. Our model, called Darwinian Neurodynamics, assumes that the unconscious mechanism of problem solving during insight tasks is a Darwinian process. It is based on the evolution of patterns that represent candidate solutions to a problem, and are stored and reproduced by a population of attractor networks. In our first experiment, we used human data as a benchmark and showed that the model behaves comparably to humans: it shows an improvement in performance if it is pretrained and primed appropriately, just like human participants in Kershaw et al. (2013)'s experiment. In the second experiment, we further investigated the effects of pretraining and priming in a two-by-two design and found a beginner's luck type of effect: solution rate was highest in the condition that was primed, but not pretrained with patterns relevant for the task. In the third experiment, we showed that deficits in computational capacity and learning abilities decreased the performance of the model, as expected. We conclude that Darwinian Neurodynamics is a promising model of human problem solving that deserves further investigation.

## Introduction

### Darwinian neurodynamics

The Bayesian brain is an increasingly popular idea in cognitive science. According to this theory, the mind assigns probabilities to hypotheses and updates them based on observations. Bayesian cognitive models were successfully used in many different areas of cognition, like learning, memory, reasoning and decision making. However, the “Bayesian brain falls short in explaining how the brain creates new knowledge” (Friston and Buzsáki, 2016), it does not account for the generation of new hypotheses; it only accounts for the selection of already existing variant hypotheses.

It has been pointed out that Bayesian update effectively implements a process analogous to selection (Harper, 2009), where the prior distribution is equivalent to an existing set of hypotheses, the likelihood function acts as the selection landscape, and the posterior distribution is the output population of hypotheses after a round of selection. If selection acts on units that can replicate and inherit their traits with variability we get full-blown evolution (Maynard Smith, 1986). We believe that the Bayesian paradigm for modeling cognition, especially problem solving, could be successfully complemented with replication and inheritance to explain where new hypotheses come from.

Problem solving can be conceptualized as search for the solution in a search space (sometimes also called the hypothesis space, state space, or problem space). The search space is the space of all hypotheses that are possible within the dimensions that the problem solver considers. Cognitive search mechanisms must be very effective in exploring the search space and must account for the generation of new hypotheses. Evolutionary search (Maynard Smith, 1986) fulfills those requirements, as it implements parallel, distributed search with a population of competing evolutionary units and it also explains the generation of these units that depends on fitness. Evolutionary search as a model for creative cognitive processes is not a new idea (see e.g., Campbell, 1960; Simonton, 1999, 2011; Fernando et al., 2012). Some of us have previously proposed (Fernando and Szathmáry, 2009, 2010; Fernando et al., 2010) the framework of Darwinian Neurodynamics (previously called the Neural Replicator Hypothesis) as a cognitive model for problem solving in the brain. In this framework, hypotheses or candidate solutions to a problem play the role of evolutionary units: they are selected based on their fitness just like in Bayesian update, but they also multiply with heredity and variation, thus the model implements a full evolutionary search and explains the generation of new hypotheses.

In de Vladar et al. (2016) and Szilágyi et al. (2016), we describe an instance of a neural implementation for a cognitive architecture and show how the synergy between selection and learning can solve pattern-matching problems. Here, we take these ideas a step further to demonstrate the problem solving capabilities of Darwinian Neurodynamics in a task that is more relevant to understanding cognition. For this purpose, we apply the Darwinian Neurodynamics framework to a classic insight task, namely, the four-tree problem.

### The four-tree problem

Insight problems are used by cognitive scientists to study insight problem solving behavior. While most agree that insight tasks can be solved analytically, these tasks usually trigger a different route of problem solving that can be characterized by typical problem solving stages, including impasse and insight (Chronicle, 2004). After an initial phase of search, when problem solving is mostly conscious and analytical, most problem solvers enter a phase of impasse when they feel that they are not getting closer to the solution (Ohlsson, 1992; Öllinger et al., 2014). Search and impasse can alternate several times (Fedor et al., 2015). While most researchers agree on the behavioral correlates of impasse (repeating previous solution attempts or becoming inactive, Ohlsson, 1992), what happens at the cognitive level remains unknown. Yet, it can be assumed that the search goes on unconsciously, because some problem solvers emerge from the impasse phase with an insight, when they figure out how to proceed.

We chose an insight task to test our cognitive architecture, because they usually have vast search spaces and their solutions are new and unusual in some sense. This is a case where evolutionary search can be very effective, because it implements parallel, distributed search and explains the generation of new hypotheses. We do not think that evolutionary search can account for all aspects of cognition, but it could have huge benefits in certain problems, where the search space is large and/or where the solution is new.

The four-tree problem is posed for participants in the following way: A landscape gardener is given instructions to plant four special trees so that each one is exactly the same distance from each of the others. How is he able to do it? (de Bono, 1967). The solution is that he plants the trees on the apices of a regular tetrahedron, so that one of the trees is on top of a hill (or at the bottom of a valley), and the other three trees are at ground level in a shape of a triangle (any other rotation of a tetrahedron would do, but this is the easiest solution in terms of the amount of landscaping that must be done).

The four-tree problem belongs to the class of 2D constraint problems (Katona, 1940; Ormerod et al., 2002), in which problem solvers implicitly impose on themselves the constraint that the problem should be solved in two-dimensional space, although the solution is three-dimensional. Most insight tasks are misleading in some way and most problem solvers unnecessarily constrain the initial search space. Restructuring (Ohlsson, 1992) happens when the problem solver, either consciously or unconsciously, lifts the constraint and starts searching in a new, unrestricted (or less restricted) search space. While these dynamics might not be true for all insight tasks (Metcalfe and Wiebe, 1987; Kershaw and Ohlsson, 2004), many other insight problems (e.g., nine-dot problem, five-square problem, ten-penny problem) can be described in this way.

We propose that the difference between conscious search and search during impasse can be modeled as search based on previous experiences vs. search during which entirely new hypotheses are generated that broaden the effective search space, respectively. We speculate that the futility of trying to solve the problem and the frustration it causes makes problem solvers to stop conscious search. This might lead to a different kind of search, which is mainly unconscious (or this might go on in parallel since before), and which might lead to restructuring. In the case of the four-tree problem, the behavioral correlate of restructuring is the appearance of the first three-dimensional solution attempt.

Kershaw et al. (2013) recently conducted a study of the four-tree problem. Their pilot work revealed that the main sources of difficulty in the four-tree problem were participants' geometric misconceptions (e.g., “believing that the diagonal of a square is the same length as the sides”) as well as their “perceptual bias of constructing a two-dimensional problem space”. In their experiments, Kershaw et al. attempted to relax the knowledge constraint with direct instructions and the perceptual constraint with analogy training. Direct instructions included teaching participants about the properties of squares, equilateral triangles and tetrahedrons. During analogy training participants had to solve three problems that were isomorphic to the four-tree problem, i.e., four objects had to be placed equidistant from each other in a shape of a tetrahedron. They conducted two experiments, which differed only in the analogy training: in Experiment 1 analogy training only posed the problems, but participants did not get feedback from the experimenter; in Experiment 2, the first two problems were presented together with their solutions, and participants were encouraged to compare these examples, then participants got feedback on their solution attempts to the third problem. Additionally, after receiving instructions for the four-tree problem half of the participants received picture clues, including pictures of trees on mountaintops, in an attempt to prime participants to think about three-dimensional landscapes and prevent unhelpful prior knowledge, activated by the task, to restrict the problem representation to two dimensions. They compared the solution rates of groups of participants who either received direct instructions, analogy training, both (combined groups) or none (control group). Experiment 1 revealed that the direct instruction and the combined groups performed better than the analogy and the control group. In Experiment 2 they found, among others, that participants with the analogy training and the combined training were more likely to solve the task than the control group and that within the combined group, participants who received picture clues were more likely to solve the task than participants who did not receive picture clues.

Kershaw et al. argue that the bias to represent the problem in two dimensions arises from prior experiences of problem solvers. We think that giving participants pen and paper to solve the problem is also a factor, in fact, it can be thought of as a misleading element in the task. We think that presenting the problem in a less misleading manner, for example asking participants to plant small model trees in a sandbox, would increase the frequency of three-dimensional solution attempts. While Kershaw et al. did not manipulate the misleading component in the task, their priming through picture clues might have influenced how much the same misleading component (i.e., giving them paper and pencil) actually misled participants.

### Motivation for the present study and predictions

#### Experiment 1

The aim of our first experiment was to benchmark the behavior of our cognitive architecture with evolutionary dynamics based on human data. Our second and third experiments provide new predictions about human behavior that are yet to be tested.

Kershaw et al. (2013)'s direct instruction training addressed gaps in prior knowledge, while their analogy training increased participants' experience with problems involving tetrahedrons. Since both training types occurred right before participants were given the four-tree problem, in our view, both served to prime participants to think about three-dimensional shapes, and particularly tetrahedrons. The picture clues can be thought of as additional and pure priming that affects the two-dimensional bias (without training), but they were only given to half of their combined training group in Experiment 2. To sum up, all their experimental groups received training with tetrahedrons and priming with tetrahedrons to some degree, while their control group received neither training, nor priming.

In our simulation experiments, we could not differentiate between the different types of trainings (direct instructions vs. analogy training), because these require higher order cognitive functions that we do not model here. Instead, we aimed at explaining the mechanistic effect of training and priming on problem solving. In our Experiment 1, we tried to reproduce the difference between the control group (1 out of 31 participants, 3% solved the problem in the given 4 min) and the combined training group with picture clues (16 participants out of 28, 57% solved the task; Kershaw, 2016, Personal communication, 28 June) in Kershaw et al.'s (2013) experiment, to provide a benchmark for our cognitive architecture (de Vladar et al., 2016; Szilágyi et al., 2016). We ran 30 simulations in both conditions and compared the problem solving behavior and performance of the models.

#### Experiment 2

In Experiment 2, we were interested in tearing apart the effects of prior experience and priming on problem solving. In a 2 × 2 design, we investigated the effects of two-dimensional vs. three-dimensional training and two-dimensional vs. three-dimensional priming. Accordingly, in the first condition, the models received two-dimensional training, and two-dimensional priming, in the second condition the models received two-dimensional training and three-dimensional priming, in the third condition, the models received three-dimensional training and two-dimensional priming and in the fourth condition the models received three-dimensional training and three-dimensional priming (we explain how these manipulations were implemented for the model in the Methods section). We ran 30 simulations in all of the four conditions, each. We predicted that the group that received two-dimensional training and priming would perform worst and that the group that received three-dimensional training and priming would perform best.

#### Experiment 3

In Experiment 3, we wanted to compare the problem solving abilities of different populations of models. Specifically, we wanted to model how different cognitive abilities might influence problem solving behavior. Chein et al. (2010) showed that a large spatial working memory capacity is beneficial for solving the nine-dot problem, another multi-step insight problem. Ash and Wiley (2006) also found that individual differences in working memory had an effect on insight problem solving. Apart from differences in working memory, we do not know of other cognitive abilities that have been investigated in connection with insight problem solving, but we assume that learning speed and synaptic efficiency could also have an effect. To investigate this question, we ran simulations with different parameter settings, one group being the control group, and three other groups representing different cognitive “deficits,” i.e., parameter settings that we think would negatively influence problem solving. These deficits were lower working memory, slower learning and less effective synapses between layers of neurons. We predicted that the deficit groups would perform worse than the control group.

## Methods

### The cognitive architecture for darwinian neurodynamics

#### Architecture of the model

Our model is also described in de Vladar et al. (2016) and Szilágyi et al. (2016). The MATLAB code of the model, the parameters and scripts for running and analyzing the experiments can be downloaded from osf.io/vjfv9.

**Figure 1:**
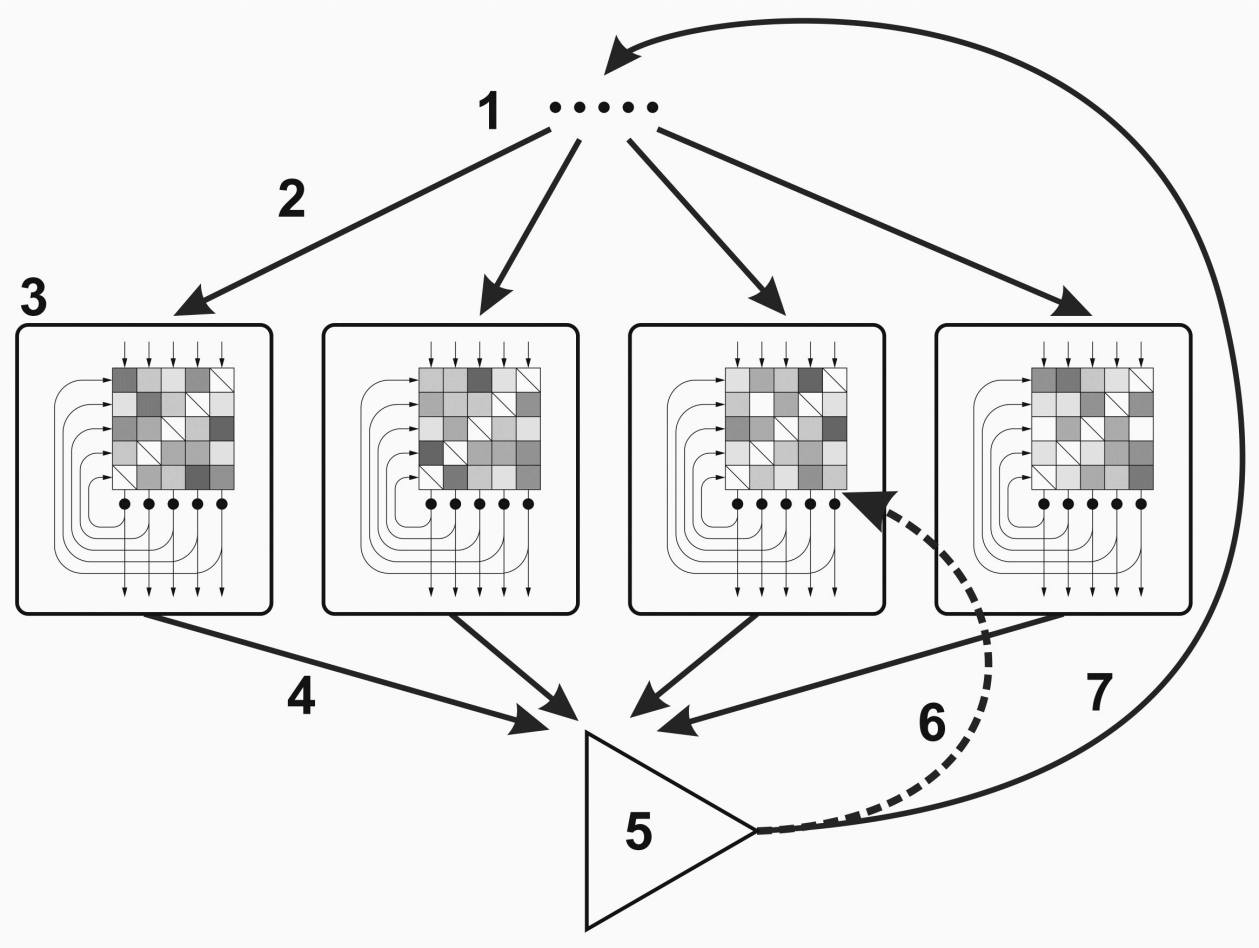
**Cognitive architecture for the Darwinian Neurodynamics theory**. (1) Selected input patterns, (2) Each network is provoked by an input pattern, (3) Attractor networks (the checkerboards represent the weight matrices; the input, output and recurrent connections are represented by arrows), (4) Output patterns are submitted to the working memory, (5) Output patterns are evaluated and the patterns with the highest fitness are selected, (6) Selected patterns are used for retraining some of the networks, (7) Selected patterns are used for provoking the networks.

The core component of our model (Figure 1) is a population of attractor networks. Attractor networks are recurrent auto-associative artificial neural networks with only one layer of units (artificial neurons). Attractor networks are fully connected, i.e., each unit is connected to all the other units within the same network (but self-connections are missing) with weighted connections (weights are real values). In these simulations, the population consisted of 100 attractor networks and each attractor network consisted of 300 units (*N* = 300).

Attractor networks can be provoked or trained with input patterns. Input patterns are binary vectors of the same length as the number of neurons in the network. When the network is trained with input pattern ξ at timestep *m*, the weight of the connection between unit *i* and *j* is calculated according to the learning rule (Storkey, 1998, 1999):

$$\begin{array}{l} w_{ij}^{m}=w_{i\;j}^{m-1}+\frac{1}{N}\xi_{i}^{m}\xi_{j}^{m}-\frac{1}{N}\xi_{i}^{m}g_{j}^{m}-\frac{1}{N}g_{i}^{m}\xi_{j}^{m}\text{ if }i\neq j,\\ w_{i\;j}^{m}=0\text{ if }i=j, \end{array}$$

with $g_{i}^{m}$ being:

$$g_{i}^{m}\;=\;\sum_{k\;=\;1}^{N}w_{i\;k}^{m-1}\xi_{k}^{m}.$$

We used a forgetting rate of *f* = 0.1, which means that the weights were multiplied by (1 − *f*) before each learning event to prevent the saturation of weights. The result of training is that the network learns (stores) the training (input) pattern. It means that when the network is later provoked (see later) with noisy versions of the training pattern, it outputs the original pattern or a pattern very similar to it (pattern completion). The learning rule we used is a modified Hebbian rule, which enables palimpsest memory (Storkey, 1998, 1999; Storkey and Valabregue, 1999), meaning that the networks can be retrained sequentially with different patterns, without inducing catastrophic forgetting. When the networks reach their memory capacity, they forget earlier patterns, but they are still able to learn new ones.

When an attractor network is provoked by a pattern, the pattern is clamped on the neurons and then the state of the neurons is recalculated according to the update rule. First, the *local field h*_*i*_ of neuron *i* is calculated as the weighted sum of recurrent signals from other neurons:

$$h_{i}=\sum_{j\;=\;1\;(\neq i)}^{N}w_{i\;j}x_{j}(t),$$

where *N* is the number of neurons in the network, *x*_*j*_*(t)* is the state of neuron *j* (active or inactive) in update step *t* and *w*_*ij*_ is the weight of the connection between neuron *i* and neuron *j*. Then, the state of neuron *i* is calculated as *x*_*i*_(*t* + 1) = sgn(*h*_*i*_). The neuron is said to be active, if its state is +1, and inactive otherwise. The state of neurons is updated asynchronously in random order (i.e., *N* neurons are chosen randomly with replacement to be updated). After *N* updates, the collective state of neurons is called the activation pattern of the network, which is a binary vector of length *N*.

The output of the neurons is then fed back as input for the next update step and the neurons are updated again. Recurrent update cycles go on until the output converges to a stable pattern or until the limit is reached (33 cycles in these experiments). The final activation pattern of the network is called the output pattern.

All networks in the model produce output patterns simultaneously. These patterns constitute one generation of output patterns. The fitness of each output pattern is then calculated by a fitness function (see later), where fitness is a real value between 0 and 1. The best patterns (patterns with the highest fitness; three patterns in these simulations) are selected and then fed back to the networks as input patterns; the rest of the patterns are deleted. Some random noise is added to the patterns during this step to simulate imperfect copying. We implemented this by randomly flipping (changing −1 to +1, and vice versa) each bit in the patterns with a probability of *m* (mutation rate).

Initializing a simulation means that we randomly generate training and provoking patterns for each network. First, each network is *pre-trained* with a different set of random patterns, i.e., each network has different weights at the beginning. Then, each network is provoked with a different random pattern and the first generation of the simulation begins. The networks produce output patterns, and the best patterns are selected based on the fitness function. The selected patterns are randomly ordered and fed back to the networks. The networks are either trained with these new patterns, or not (see later). If training happens, it is called *retraining*, to differentiate it from the initial *pre-training*. The selected patterns are randomly ordered again to provoke the networks and the second generation of the simulation begins. The simulation goes on until one of the selected patterns reaches fitness = 1 or until time out.

#### Evolution and selection modes

As we mentioned above, input patterns are either used to retrain some of the networks or not. We call these two different working modes of the model evolution mode and selection mode, respectively. In evolution mode, networks can be retrained with the selected patterns with a probability of *r* (retraining probability). The term “evolution mode” makes sense, if we consider that in this mode the whole system effectively implements evolutionary search for the pattern with the highest fitness.

Evolutionary units have three essential traits: multiplication, inheritance and variability (Maynard Smith, 1986). In a population of evolutionary units, if these units are multiplied with variation and if their hereditary traits influence their fitness, evolution takes place. In our model the evolutionary units are the patterns. In each step of the simulation, a new generation of output patterns are produced by the attractor networks. Output patterns are similar to the input provoking patterns if a similar pattern is stored in the network. This step implements inheritance with variability. A few patterns are selected based on their fitness and these are copied with errors (mutations) back to the attractor networks as inputs. These patterns multiply when they are used as retraining inputs. They get stored in more networks, which in turn will be able to reproduce these patterns if they are provoked with a correlated pattern.

There are several sources of variation of patterns in this architecture. The first one is a result of the stochastic asynchronous update of the attractor networks. This means that an attractor network usually produces slightly different output patterns when repeatedly provoked by the same input. Second, each attractor network in the population has a unique training history, thus they produce different outputs when provoked with the same input. Third, copy connections are error-prone, i.e., when the selected patterns are copied back to the networks to provoke and to retrain them, they go through mutations. Finally, networks sometimes produce so called spurious patterns, which are different from any of the previously trained patterns or even the input pattern. This usually happens when the input pattern is quite far from the training patterns, thus none of the stored patterns can be retrieved.

In evolution mode, the model performs evolutionary search, and it can be thought of as an evolutionary algorithm in the sense that it is “based on the model of natural evolution as an optimization process” (Bäck et al., 1993). The attractor networks take care of multiplication with inheritance and variation. The selected patterns are copied back with errors to the networks as inputs through neural afferents. These are the components that are neurally implemented, while the fitness function and selection mechanism are symbolic. One novelty of the model is that in fact, it is possible to semi-neurally implement evolutionary search through a population of attractor networks. Inheritance is different from that of other evolutionary algorithms because selected patterns are not directly replicated but instead trained to networks which can in turn reproduce them. Our experiments show that this kind of indirect replication results in evolutionary dynamics similar to that of asexual populations of evolutionary units (there is no cross-over).

In selection mode, the networks are not retrained and thus their output is solely dependent on their pretraining and on the input (provoking) pattern. We call this selection mode, because it is based purely on selection over the standing variation: the best patterns are selected but they do not reproduce, they do not spread to new networks. Because of this, the model can only search in the space of already available patterns and their close neighbors (there is still mutation during copying of provoking patterns).

In selection mode, our model is similar to Bayesian cognitive models of learning and problem solving, where output patterns play the role of hypotheses (Griffiths et al., 2010; Tenenbaum et al., 2011), because Bayesian update is analogous to selection (Harper, 2009) as we described in the Introduction.

#### Problem solving as evolutionary search

We think of this model as a cognitive process model for problem solving which is also neurally plausible to some extent. Patterns represent hypotheses, or candidate solutions to a problem that a problem solver might entertain during problem solving. Patterns are either stored in the long-term memory represented by the weight matrices of attractor networks or in the working memory that consist of the maintained activation of the networks. We call the pattern with the highest fitness in each generation a candidate solution. We suggest that most hypotheses are unconscious and only a small sample emerges into consciousness. Solution attempts are candidate solutions that the problem solver acts out, i.e., draws on the given paper or describes verbally. They allow us a very limited peek into the thought processes of participants in insight experiments. We propose that human participants sample their solution attempts from the candidate solutions, and only a small subset of the candidate solutions become conscious, especially, during impasse. Human participants probably generate new hypotheses at different rates, but for the sake of simplicity, we equate generations of patterns in the model with time steps.

We conceptualize priming as an effect on the initial assumptions of the problem solver. These initial assumptions are modeled by the first set of patterns by which the attractor networks are provoked before the first generation of output patterns emerges. By manipulating how these initial provoking patterns are generated, we can model different priming conditions.

Pre-training patterns are analogous to prior experiences of problem solvers and possible solutions to problems that are stored in long-term memory. Selection and evolution modes model two different thinking modes in humans: selection mode is when the problem solver searches for the solution in long-term memory and evolution mode is when the problem solver generates new hypotheses.

When solving insight tasks, humans first try to solve the problem based on their previous experiences (selection mode). Insight tasks are constructed in a way that previous experiences combined with some misleading elements in the task drive problem solvers to unnecessarily restrict the search space. For example, when the four-tree problem is presented on a piece of paper, it misleads participants to think that the solution must be two-dimensional. This coincides with the fact that most people have more experience in two-dimensional paper-and-pencil type tasks than in three-dimensional tasks. To find the solution, problem solvers need to switch to a different thinking mode, where they consider new hypotheses (evolution mode). This might lead to extending their search space to three dimensions through representational change (restructuring).

To model this process, we start simulations in selection mode and then switch to evolution mode with a certain probability. Before the switch between modes, the model only searches based on its previous experiences, whereas after the switch, new candidate solutions can evolve. Without the switch, finding a solution is only possible if long-term memory already contained the solution. We implement switching in a probabilistic way so that it can occur any time during problem solving with a certain probability. The probability of switching is calculated in each generation of patterns by the following equation:

$$s\;=\;1/r^{c}{\;}^{*}\;(1-a^{b\;*\;g}),$$

where *r* is the number of repeated candidate solutions so far, *g* is the number of generations so far, and *a, b*, and *c* are constants, which were set to 0.7, 0.03, and 1.0, respectively. We suggest, that these parameters can be adjusted when the architecture is used to model different tasks. Switching happens only once during a simulation, which is a simplification. We plan to implement back-and-forth probabilistic switching in our future work.

As indicated, the first term of the equation (1/*r*^*c*^) is dependent on the number of repeated candidate solutions. The probability of switching decreases as the number of repetitions increases and selection mode also increases the probability of producing a repeated candidate solution. Repeated candidate solutions are patterns that represent solutions to the problem that has already occurred in a previous generation. It has been shown (Kershaw et al., 2013; Fedor et al., 2015) that in human problem solvers the number of repeated solution attempts is inversely proportional to the probability of solving the task. In fact, repetitions are one of the two behavioral associates of impasse. One possibility is that repetitions cause impasse as a self-induced mental set (Luchins, 1942; Lovett and Anderson, 1996; Öllinger et al., 2008). A second possibility is that repetitions are a direct consequence of either a saturated working memory (the problem solver forgets that he has already tried a solution attempt) or an inability to generate new hypotheses, which makes it less probable that a solution is found. The first term of the switching probability equation implements a causative relationship between repetitions and the inability of getting out of impasse. However, the other factors, namely a poor working memory, is also present indirectly (see Experiment 3).

**Figure 2:**
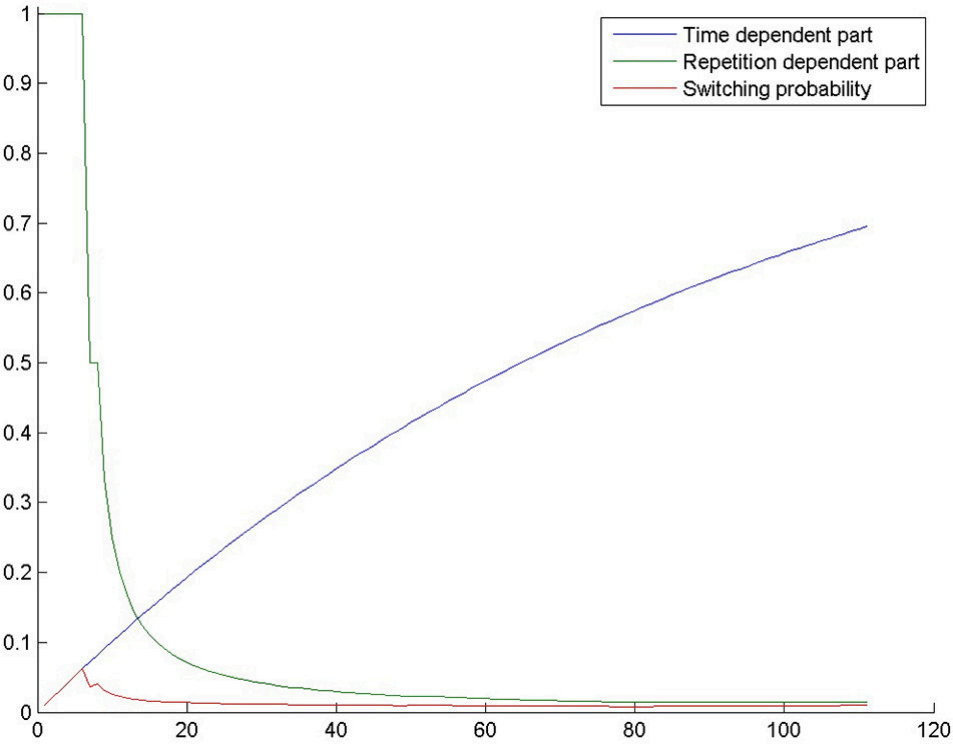
**The probability of switching from selection mode to evolution mode in a simulation in the control condition of Experiment 2**. The blue curve shows 1−*a*^b^*^g^, where g is the number of generations, *a* = 0.7 and *b* = 0.03. The green curve shows 1/*r*^*c*^, where r is the number of repetitions and *c* = 1. The red curve is switching probability, which is the product of the previous terms.

The second part of the equation (1−a^b^*^g^) is proportional to the number of generations, i.e., it is proportional to the time spent by trying to solve the task. We assume that as time passes, problem solvers become more likely to realize that their initial search space is insufficient and that they need to look for a solution in a different search space. Figure 2 shows the probability of switching through the generations in one of our simulations. It can be seen that if the model fails to switch in the first few tens of generations, switching becomes quite improbable.

### Implementing the four-tree problem

#### Adaptation of the task for the model

**Figure 3:**
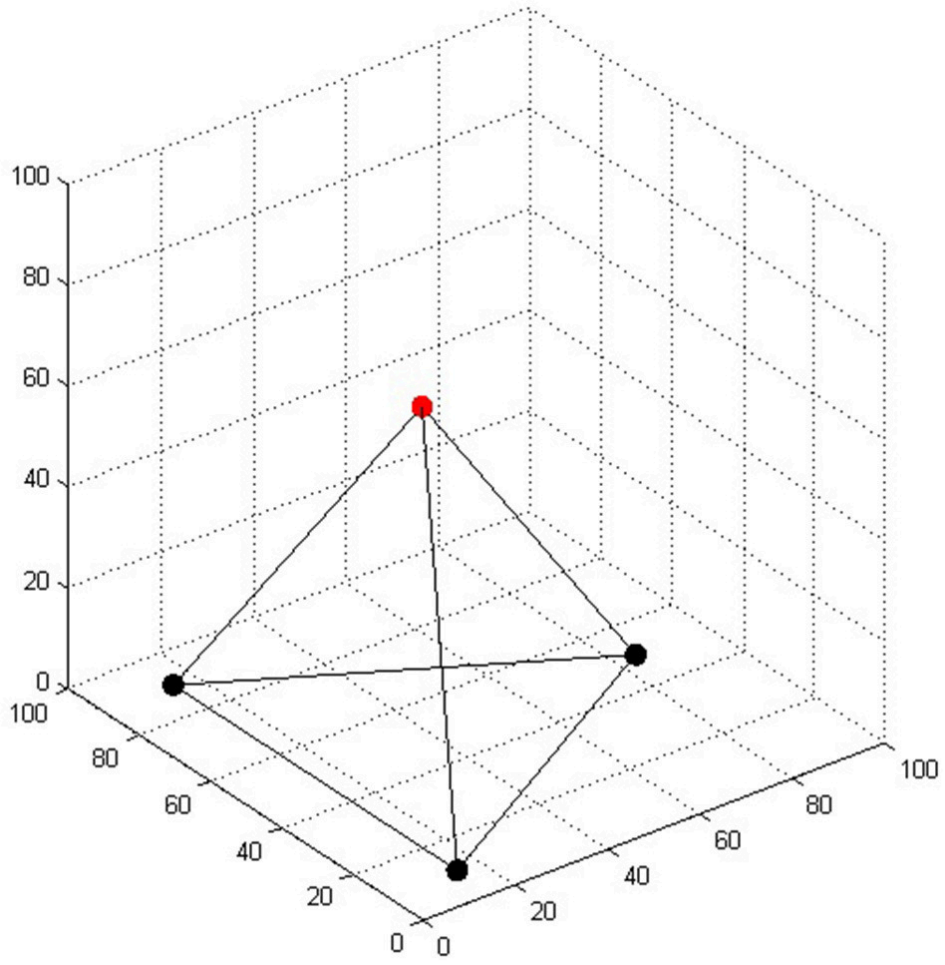
**The regular tetrahedron in the four-tree problem**. The three bottom trees represented by black dots were already “planted”; the task of the model was to place the fourth tree represented by the red dot.

In the original four-tree problem the task of the landscaper is to plant all four trees. Here, we modified this task so that only one of the trees must be placed; the rest of the trees are already planted in a shape of a triangle on a plain surface (Figure 3). While there have not been human experiments with this modification, we can safely assume that the main problem difficulty (the two-dimensional bias) remains the same. We represented the trees in a three-dimensional coordinate system, where each axis ranged from 0 to 100. The distance between each pair of trees was 80 units. The coordinates of the four trees were rounded to the nearest integer: (15, 10, 0), (15, 90, 0), (84, 50, 0), and (38, 50, 65). The last set of coordinates represents the fourth tree that the model has to place in order to solve the task.

#### Representation of the task

An important aspect of modeling problem solving behavior is how to translate the human-readable puzzle to a problem defined within the model and how to translate the outputs of the model to candidate solutions. The output patterns of attractor networks are necessarily binary patterns so we need a representation where these patterns (300-bit-long binary vectors) can be unambiguously converted to a point in space where the fourth tree is placed.

This conversion should take into consideration the properties of attractor networks. For example, attractor networks have probabilistic outputs, i.e., they can have slightly different outputs when provoked with the same input. Because of this, slight differences in the output should not translate to major differences in the candidate solution: outputs that only differ in a few bits should represent points in space that are close to each other. A cumulative conversion, where the number of active neurons (or the sum of the vector) is proportional to some kind of effort or movement that the efferent of the system (that we do not model here) exhibits in order to place the tree, seems to be a natural way of representing this problem.

The conversion that we used is very simple: the output patterns of networks represented the *x, y*, and *z* coordinates of the fourth tree in the following way:

- x coordinate = number of active neurons from neuron 1 to neuron 100,
- y coordinate = number of active neurons from neuron 101 to neuron 200, and
- z coordinate = number of active neurons from neuron 201 to neuron 300.

If we put the fourth tree in the (0,0,0) position before each solution attempt, the output pattern can be interpreted as an instruction about moving the tree to its final position. The number of active neurons equals to the number of units of movement in the three dimensions. While this representation is probably not how a location in three-dimensional space is represented in the brain, the details of the model are not essential to the evolutionary argument.

#### Fitness function

The fitness of patterns was based on the hypothetical instructions (“Plant the fourth tree so that it is the same distance from all other trees as they are from each other”): how close is the distance of trees to the target distance:

$$\begin{array}{l} \text{Fitness }=\;1\;-(\text{sum}(\text{abs}(\text{round}(\text{distance}\\ \;({\text{tree}}_{1-3},\;{\text{tree}}_{4}))-\text{target})/3^{*}\text{target}), \end{array}$$

where tree_1−3_ are the already planted trees, tree_4_ is the tree whose coordinates the model has to find, and target is the target distance between trees (target = 80 in these simulations).

#### Initializing simulations

Pre-training patterns and initial provoking patterns were generated probabilistically with three different sparseness values representing the probability that a unit responsible for the *x, y*, or *z* coordinate is active. For example, a sparseness of [0.5, 0.5, 0.0] means that within a pattern, each *x* and *y* neuron has a state of +1 with a probability of 0.5 and −1 with a probability of 0.5, while all *z* neurons are inactive. Within each simulation, 90 pre-training-patterns and 1 initial pattern was generated for each attractor network. The sparseness of these patterns differed across conditions.

### Simulation experiments

#### Experiment 1

In this experiment, we simulated the positive effects of training and priming on solution rates. We wanted to reproduce the results of Kershaw et al. (2013), more specifically, the difference between their control group and their combined group with picture clues. We ran two groups of simulations, where each simulation can be thought of as one individual in the experiment. The combined condition received pre-training in two-dimensions and on tetrahedrons and priming on tetrahedrons; the control condition received pre-training and priming in two-dimensions.

The question might arise why we pre-trained the control group at all. In simulations, we have to simulate participants' previous experiences (i.e., their “training” that happened throughout their lives, before they arrived to the experiment) and also the training that they might receive as an experimental manipulation. Human participants who do not receive training during the experiment are left with their previous experiences, which we suppose are predominantly two-dimensional regarding paper-and-pencil type tasks, because most people do not solve three-dimensional tasks very often (this might be one of the reasons for the low solution rates in the four-tree problem). These predominantly two-dimensional experiences are modeled as pre-training with two-dimensional patterns in our simulations. These patterns were generated with a sparseness of [0.5, 0.5, 0.0] (90 pre-training patterns for each network), which meant to represent general two-dimensional experiences.

The combined group received both two-dimensional pre-training (sparseness = [0.5, 0.5, 0.0] for 45 patterns), and pre-training on patterns representing tetrahedrons (sparseness = [0.38, 0.50, 0.65] for 45 patterns). This pre-training regime modeled that participants in the combined condition had similar two-dimensional experiences as the control group, but they were trained with exercises involving tetrahedrons before they were given the main task.

We conceptualized successful priming as an effect on participants' initial hypotheses about the task. This is a starting point for subsequent hypotheses, as it initializes the thought process. Successful priming with tetrahedrons results in initial hypotheses that are close to tetrahedrons. No priming means that the misleading presentation of the task takes over, and the initial hypotheses are two-dimensional. In this sense, we can think of the control group in Kershaw et al.'s (2013) experiment as a group that received two-dimensional priming in the form of the misleading presentation of the task. To reflect this difference, our control group was “primed” (initialized) with two-dimensional patterns (sparseness = [0.38, 0.5, 0.0]), and the combined group was initialized with patterns representing tetrahedrons (sparseness = [0.38, 0.50, 0.65]). The sparseness of the initializing patterns for the control group was derived from the coordinates of the already planted three trees: the *x, y*, and *z* sparseness values were calculated as the averages of the *x, y*, and *z* coordinates of the trees. This meant to model that when there is no deliberate priming, participants draw their initial assumptions from the presentation of the task.

In both conditions, we ran 30 simulations, initialized with the same random seed across conditions, to be able to easily compare our results with the results of Kershaw et al. (2013) who had 31 participants in their control condition and 28 participants who received combined training and picture clues.

#### Experiment 2

In this experiment, we investigated the effect of prior experiences and priming in a two-by-two design: Table 1 shows the resulting four conditions.

**Table 1:** **Treatment conditions in Experiment 2**.

	**Initialized with 2D provoking patterns derived from the task presentation: sparseness = [0.38, 0.5, 0]**	**Initialized with 3D random provoking patterns independently of the task presentation: sparseness = [0.5, 0.5, 0.5]**
Pre-trained on 2D random patterns: sparseness = [0.5, 0.5, 0]	**Condition 2DD** (2D pre-training + Derived initial patterns)	**Condition 2DR** (2D pre-training + Random initial patterns)
Pre-trained on 3D random patterns: sparseness = [0.5, 0.5, 0.5]	**Condition 3DD** (3D pre-training + Derived initial patterns)	**Condition 3DR** (3D pre-training + Random initial patterns)

Condition 2DD (**2D** pre-training, **D**erived patterns for initializing) was identical to the control condition in Experiment 1 (but initialized with different random seeds): it was pre-trained with two-dimensional patterns (sparseness = [0.5, 0.5, 0]) and initialized with two-dimensional patterns derived from the task (sparseness = [0.38, 0.5, 0], calculated as the averages of the coordinates of the three planted trees).

Condition 2DR (**2D** pre-training, **R**andom patterns for initializing) received the same two-dimensional pre-training patterns (sparseness = [0.5, 0.5, 0]) as condition 2DD, but was initialized with three-dimensional patterns with sparseness = [0.5, 0.5, 0.5]. These patterns model the result of either priming with three-dimensional shapes, or a less misleading presentation of the task (sandbox).

Condition 3DD (**3D** pre-training, **D**erived patterns for initializing) was pre-trained with three-dimensional patterns (sparseness = [0.5, 0.5, 0.5]) and initialized with two-dimensional patterns derived from the task (sparseness = [0.38, 0.5, 0], just like condition 2DD).

Condition 3DR (**3D** pre-training, **R**andom patterns for initializing) was pre-trained with three-dimensional patterns (sparseness = [0.5, 0.5, 0.5]) and initialized with three-dimensional patterns (sparseness = [0.5, 0.5, 0.5]). In some sense, this condition is similar to the combined condition of Experiment 1 as both training and priming were three-dimensional, but both manipulations were weaker (meaning, probably less effective in increasing performance compared to the control condition). Here, three-dimensional pre-training involved general three-dimensional patterns, not tetrahedrons as in Experiment 1 and was not mixed with two-dimensional patterns. Three-dimensional priming was also more general than in Experiment 1, because it did not involve tetrahedrons *per se*, but general three-dimensional patterns.

In each condition, we ran 30 simulations. Simulations were initialized with the same random seed across conditions, thus conditions can be thought of as repeated manipulations on the same group of participants (but the effects of previous conditions erased).

#### Experiment 3

In this experiment, we modified some parameters of the model in a way that we suspected to cause a deficit in the problem solving abilities of the model. The result of deficits could be lower probability of solving the problem, or slower problem solving. These modifications model the problem solving abilities of different human problem solvers.

To model these differences, we ran simulations in four different groups of models. The control group (CC) had identical parameters to the control condition in Experiment 1, but was initialized with a different random seed. In each of the other three groups one of the default parameters was changed (all the default parameters can be seen at the repository link given at the beginning of the methods section). The MC group (Memory Capacity) had a lower memory capacity: the number of attractor networks was 10 instead of 100. The MR (Mutation Rate) group had 10 times higher mutation rate (0.3 instead of 0.03) on the copying connections than the CC group. The RR group (Retraining Rate) had 10 times lower retraining probability than the control group (0.07 instead of 0.7).

Similarly to the previous experiments, in each group we ran 30 simulations, initialized with the same random seed.

### Analysis

Each simulation was run for a maximum of 200 generations, i.e., 200 subsequent candidate solutions were selected. This timeframe was chosen because our previous simulations showed that in most simulations, fitness reached a plateau by this point. We do not assert that this timeframe is equivalent to the time limit given to human participants in experiments, for example, 4 min in Kershaw et al.'s experiment (Kershaw et al., 2013). We do not know of any study that measures how human solution rates change with time in a more extended timeframe, but we speculate that 200 generations are equivalent to several hours of thinking time in humans. A simulation was scored as a “solver” if the model found the correct position for the fourth tree within this timeframe. We would like to point out that by setting a time out, we turn a possibly quantitative difference between individuals (the speed of problem solving) into a qualitative difference (solver vs. non-solver). To make our results more comparable to human data, we also calculated solution rates at the time point when the first solver appeared in the control condition, because that is how many people solved the task in the control condition of Kershaw et al. (2013) within 4 min.

We also looked at the time spent with the task, measured as the number of generations that the model went through until it either solved the task, or it reached time out. In the former case, time spent with the task equals solution time, in the latter case, time spent with the task equals time out (200 generations). Of course, we cannot assume, that every person comes up with new candidate solutions at the same rate, but this is a simplification we made, because we did not want to overcomplicate the model at this initial stage by modeling time. Time spent with the task can be broken down to selection phase and evolution phase. Selection phase starts with the first generation and lasts until switching to evolution phase. Evolution phase starts from the switch and lasts until the model either solved the problem or reached time out.

Since simulations in each condition were initialized with the same random seed within experiments, conditions can be thought of as different treatments given to the same group of individuals. Thus, we used repeated measures statistics to compare time spent with the task, the length of selection phase and the length of evolution phase. The data in one or more conditions were not normally distributed so we used nonparametric tests.

We also looked at the number of repetitions. A repetition is a candidate solution that has already been selected before. It is a repetition of the coordinates of the fourth tree, not a repetition of output patterns, i.e., many output patterns can code the same coordinates.

Finally, we also looked at the dimensions of candidate solutions. The interesting questions is whether three-dimensional candidate solutions are present from the beginning, or they only appear later during problem solving. If candidate solutions are three-dimensional from the beginning, it means that the problem solver did not need representational change, because the initial search space was already three-dimensional.

## Results and discussion

### Experiment 1

#### Number of solvers

**Figure 4:**
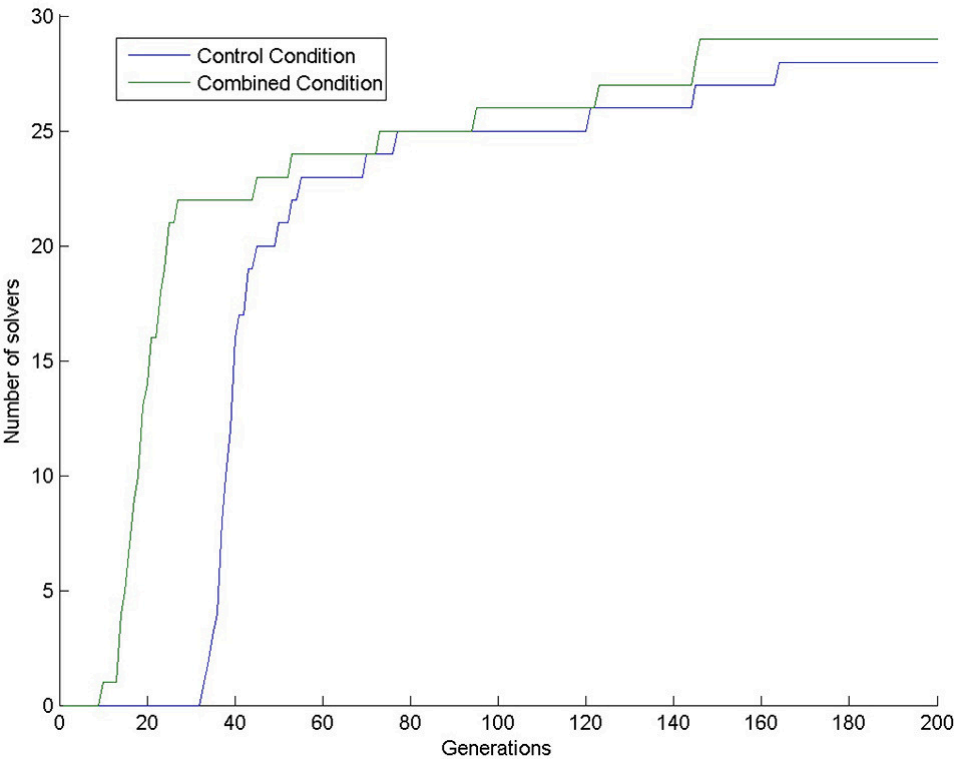
**Number of solvers through time (generations) in the two conditions of Experiment 1**.

Almost all simulations found the solution: 28 in the control condition (out of 30) and 29 in the combined condition (also out of 30). This means that 200 generations are too long compared to the 4 min given to human participants, because only one human participant (out of 31 participants; 3.2%) solved the task in the control condition (Kershaw et al., 2013) in the given 4 min. In our simulations, the first solution (3.3%) in the control condition appeared at generation 33. In the combined condition, there were already 22 solutions by that time (73.3%), see Figure 4. We compared the number of solvers in the two conditions at generation 33 with a chi-square test and we found a significant interaction: $\chi_{\left(1\right)}^{2}$ = 28.202, *p* < 0.0001.

#### Dimension of candidate solutions

When we looked at the candidate solutions, we found that all simulations in the control condition had two-dimensional candidate solutions at the beginning, and successful problem solvers later started to use three-dimensional patterns. In contrast, all simulations in the combined condition used three-dimensional patterns from the very beginning. This means that priming and pre-training with three-dimensional patterns removed the bias to solve the task in two dimensions. We have no comparable data from the human experiment.

### Experiment 2

#### Number of solvers

The number of solvers was 25, 29, 26, 23 in the 2DD, 2DR, 3DD, and 3DR conditions out of 30 simulations, respectively. According to the χ^2^ test the row and column variables are not significantly associated in the contingency table: χ^2^(df = 3) = 5.140, *p* = 0.1618.

**Figure 5:**
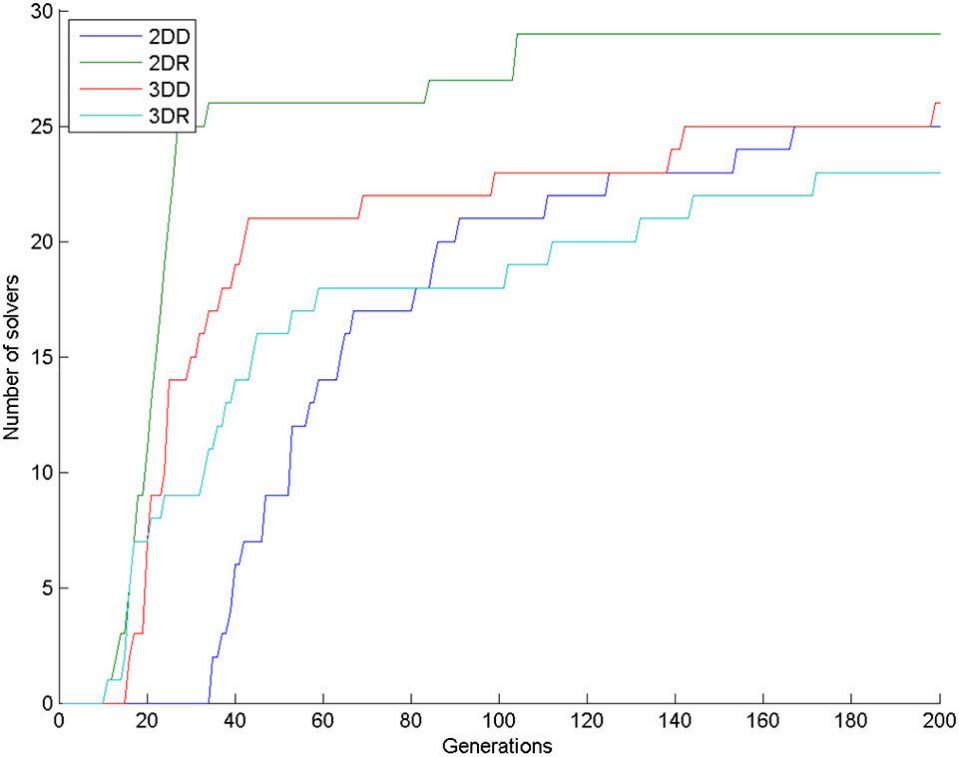
**Number of solvers through time (generations) in the four conditions of Experiment 2**.

Looking at the number of solvers through time (Figure 5) shows that earlier differences between conditions tend to disappear halfway through the simulations, except for condition 2DR, which always has the highest number of solvers. To reveal earlier differences, we also compared the number of solvers at the time point, where the first solver appeared in the control condition. This happened in generation 35, when the number of solvers was 2, 26, 17, 11 in the 2DD, 2DR, 3DD, and 3DR conditions out of 30 simulations, respectively. According to the χ^2^ test the row and column variables are significantly associated in the contingency table: $\chi_{\left(3\right)}^{2}$ = 40.982, *p* < 0.0001.

The number of solvers at generation 35 shows an unexpected rank order: 2DD < 3DR < 3DD < 2DR. Table 2 shows the results of pair-wise comparisons with a series of six χ^2^ tests. We used Bonferroni correction to compensate for multiple comparisons: α = 0.05/6 = 0.0083. The difference between consecutive conditions in the rank order was not significant, but all other differences were significant. The 2DD condition had the least number of solvers, as we predicted, but the order of the 3DR and 2DR conditions were swapped compared to our predictions.

**Table 2:** **Results of pair-wise comparisons with a series of χ^2^ tests on the number of solvers at generation 35 in Experiment 2**.

**Comparison**	**2DD (2/30)**	**3DR (11/30)**	**3DD (17/30)**	**2DR (26/30)**
2DD	−	ns	$\chi_{\left(1\right)}^{2}$ = 15.096, *p* < 0.0001	$\chi_{\left(1\right)}^{2}$ = 35.424, *p* < 0.0001
3DR	−	−	ns	$\chi_{\left(1\right)}^{2}$ = 13.819, *p* = 0.0002
3DD	−	−	−	ns
2DR	−	−	−	−

#### Length of selection and evolution phases

**Figure 6:**
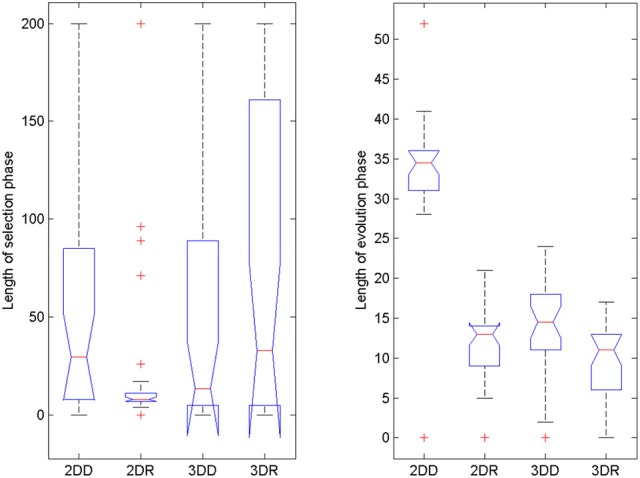
**Length of selection phase and evolution phase in the four conditions of Experiment 2**. On each box, the central mark is the median, the edges of the box are the 25 and 75 percentiles, the whiskers extend to the most extreme data points not considered outliers and outliers are plotted individually (red +). Notches represent comparison intervals: two medians are significantly different at the 5% significance level if their intervals do not overlap.

To reveal what could have caused superior performance in the 2DR condition, we checked when the switch between selection mode and evolution mode happened and how long each phase took (Figure 6). The models were not pre-trained with the solution, so finding the solution without switching to evolution mode was very unlikely. It seems that the 2DR group performed better than expected, because only one simulation did not switch to evolution mode (it is the outlier in the figure, for which the evolution phase was 0 generations long). In the 2DD, 3DD, and 3DR conditions, 4, 3 and 6 simulations failed to switch. Figure 6 also shows that most simulations in the 2DR condition switched very early to evolution mode, whereas the time of switching is more widely spread in the other conditions.

For the length of the selection phase, according to the Friedman test, variation among condition medians is significantly greater than expected by chance, Fr = 8.883, *p* = 0.309, but pairwise comparisons with Dunn's multiple comparisons test did not show significant differences between conditions. For the evolution phase, the Friedman test was also significant, Fr = 37.653, *p* < 0.0001, and Dunn's pairwise comparisons showed that the evolution phase in the 2DD condition was significantly longer than in the other conditions (rank sum difference was 45.5, 38.5, and 56.0 for 2DD vs. 2DR, 2DD vs. 3DD, and 2DD vs. 3DR, the *p* < 0.001 for all three comparisons), and there were no other significant differences between conditions.

#### Number of repetitions

**Figure 7:**
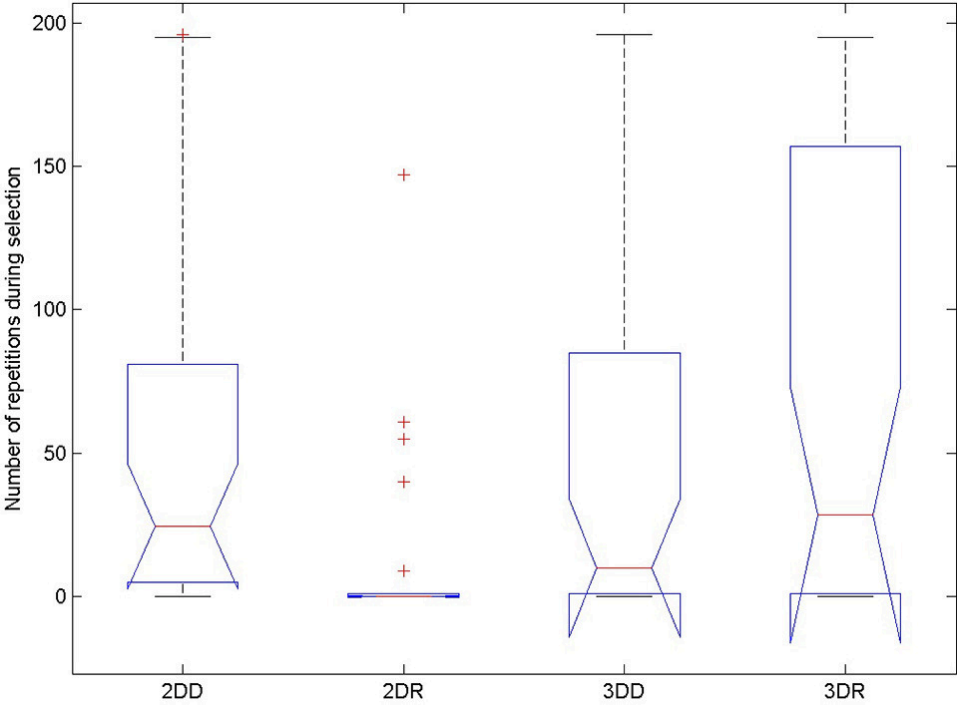
**Number of repeated candidate solutions during the selection phase in the four conditions of Experiment 2**. On each box, the central mark is the median, the edges of the box are the 25 and 75 percentiles, the whiskers extend to the most extreme data points not considered outliers and outliers are plotted individually (red +). Notches represent comparison intervals: two medians are significantly different at the 5% significance level if their intervals do not overlap.

The probability of switching depends on the number of repetitions before the switch, so we compared the number of repeated candidate solutions during the selection phase in the four conditions to see whether this could have caused the advantage of the 2DR condition, see Figure 7 and Table 3. According to the Friedman test, variation among column medians was significantly greater than expected by chance, Fr = 28.093, *p* < 0.0001. Dunn's multiple comparisons test showed that condition 2DR had significantly fewer repetitions than the other conditions, and there were no more significant differences between conditions.

**Table 3:** **Results of Dunn's multiple comparisons test on the number of repeated candidate solutions during the selection phase in Experiment 2**.

**Comparison**	**Rank sum difference**	***p*-value**
2DD vs. 2DR	42.000	*p* < 0.001
2DD vs. 3DD	4.000	ns
2DD vs. 3DR	2.000	ns
2DR vs. 3DD	−38.000	*p* < 0.001
2DR vs. 3DR	−40.000	*p* < 0.001
3DD vs. 3DR	−2.000	ns

**Figure 8:**
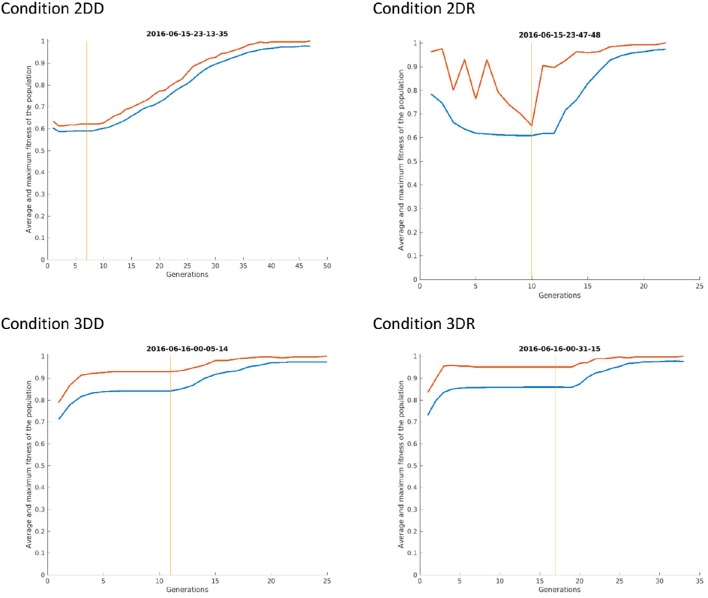
**The first simulation from each condition of Experiment 2**. The red curve shows the maximum fitness (i.e., the fitness of the candidate solution) and the blue curve shows the average fitness of all patterns in the given generation. The vertical line indicates the time of switching from selection mode to evolution mode.

This means, that the advantage of condition 2DR came from earlier switching to evolution mode because of very few repetitions. Probably the weight matrix trained on two-dimensional patterns and then provoked with three-dimensional patterns resulted in very hectic behavior, where the selected patterns of subsequent generations were very dissimilar. This is because the provoking patterns were very far from the attractor basins of the networks so that the output was more or less random, until evolution was switched on. Figure 8 shows the first simulation from each condition: it can be seen that in condition 2DR the fitness is very variable at the beginning, compared to the other conditions. The reason for condition 3DR performing worse than expected is the opposite: the interaction of three-dimensional pre-training and three-dimensional initial provoking patterns resulted in too uniform candidate solutions and many repetitions, thus late switching to evolution. Even though the initial fitness was the highest among conditions, late switching resulted in inferior performance.

#### Dimensions of candidate solutions

We also looked at the dimensions of candidate solutions. All simulations in the 2DD condition started with two-dimensional candidate solutions, whereas the rest of the conditions had three-dimensional candidate solutions from the very beginning. This explains why evolution phase in the 2DD condition was longer than in the other conditions: because when evolution started, candidate solutions were still two-dimensional, and it took longer to gather activations in the *z* coordinate starting from 0 through mutations than in the other conditions, where the *z* coordinate was already a higher than 0 value at the time of switching.

### Experiment 3

#### Number of solvers

**Figure 9:**
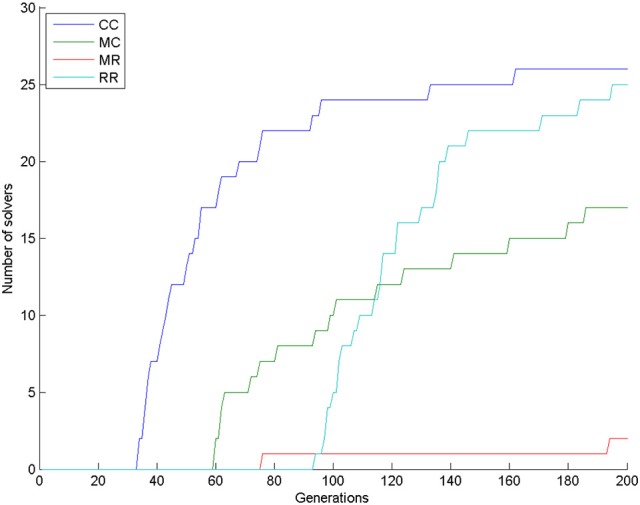
**Number of solvers through time (generations) in the four conditions of Experiment 3**.

The number of solvers after 200 generations was 26, 17, 2, and 25 in the CC, MC, MR, and RR groups. According to the χ^2^-square test, the row and column variables are significantly associated in the contingency table: χ^2^(df = 3) = 50.606, *p* < 0.0001. Figure 9 shows that group CC had the most solvers at all generations as expected, group MC was the second until about generation 110, when group RR caught up with it, and group MR had the least number of solvers most of the time.

#### Time spent with the task

**Figure 10:**
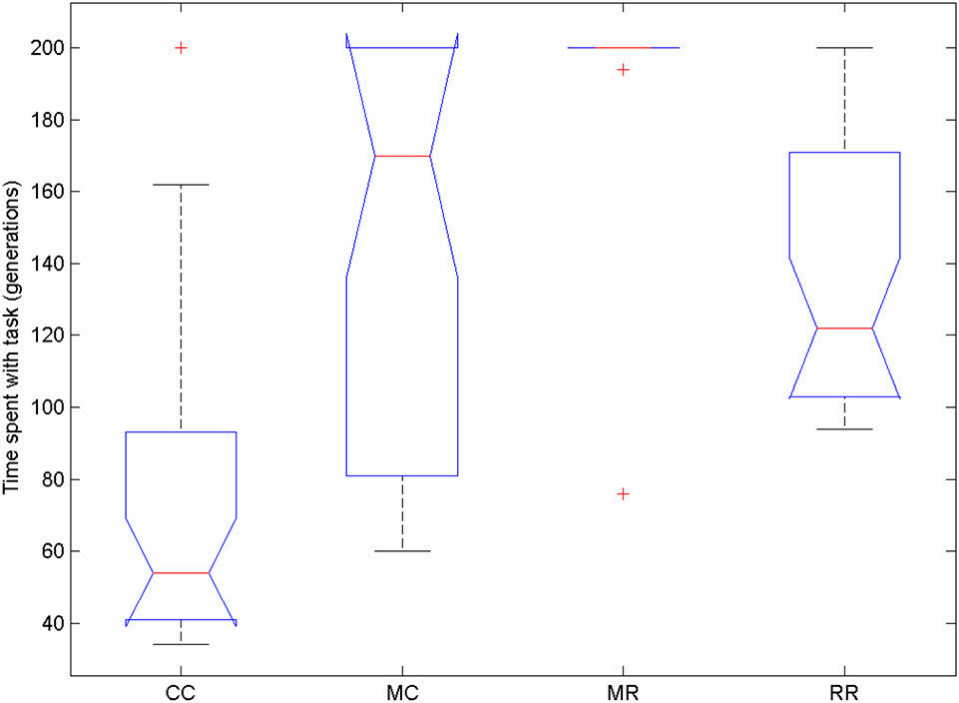
**Time spent with the task in the four conditions of Experiment 3**. On each box, the central mark is the median, the edges of the box are the 25 and 75 percentiles, the whiskers extend to the most extreme data points not considered outliers and outliers are plotted individually (red +). Notches represent comparison intervals: two medians are significantly different at the 5% significance level if their intervals do not overlap.

We compared the time spent with the task in the four conditions (Figure 10). We used Friedman test because the data were not normally distributed and then compared all groups to the control group with Dunn's multiple comparisons test. The Friedman test showed that variation among group medians is significantly greater than expected by chance, Fr = 54.477, *p* < 0.0001. Pairwise comparisons showed significant difference between the control group and all deficit groups, see Table 4.

**Table 4:** **Results of Dunn's multiple comparisons test on the time spent with the task and on the length of evolution phase in Experiment 3**.

**Comparison**	**Task time**		**Evolution phase**	
	**Rank sum difference**	***p*-value**	**Rank sum difference**	***p*-value**
CC vs. MC	−39.000	*p* < 0.001	−14.000	ns
CC vs. MR	−69.000	*p* < 0.001	−66.500	*p* < 0.001
CC vs. RR	−38.000	*p* < 0.001	−41.500	*p* < 0.001

#### Length of selection and evolution phase

**Figure 11:**
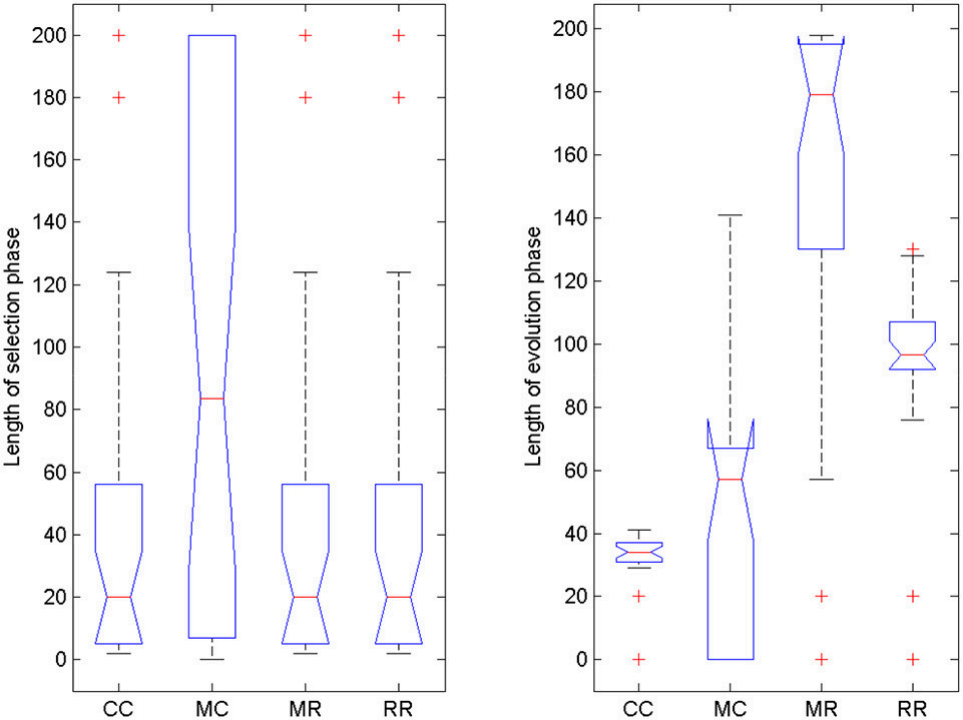
**Length of selection phase and evolution phase in the four conditions of Experiment 3**. On each box, the central mark is the median, the edges of the box are the 25 and 75 percentiles, the whiskers extend to the most extreme data points not considered outliers and outliers are plotted individually (red +). Notches represent comparison intervals: two medians are significantly different at the 5% significance level if their intervals do not overlap.

We also compared the length of selection and evolution phase between groups, as in Experiment 2, see Figure 11. Since mutation rate and retraining rate did not influence the simulations in the selection mode, all simulations in the CC, MR and RR groups were identical until evolution switched on. That is why we only compared the length of the selection phase in the control group and group MC. The Wilcoxon matched-pairs signed ranks test showed that the median of the differences between the two groups differed significantly from zero: W = −185, *p* = 0.0176. For the evolution phase, the Friedman test showed that variation among group medians was significantly greater than expected by chance, Fr = 56.740, *p* < 0.0001, and pairwise comparisons with Dunn's multiple comparisons test between the deficit groups and the control group revealed that the MR and RR groups spent more time with the task in the evolution phase than the control group, see Table 4.

## Conclusions

### Summary of results

We developed a model for human problem solving that is based on the selection and evolution of hypotheses (de Vladar et al., 2016; Szilágyi et al., 2016). The model is a possible cognitive architecture for Darwinian Neurodynamics and it is based on a population of attractor networks that store and reproduce the hypotheses which are then selected for reproduction according to their fitness. We assumed that search for the solution starts in a computationally cheaper selection mode, when the model only explores previously learnt candidate solution patterns. If the model has not met the given task before, selection generally does not find the solution. If the model switches to evolution mode, it can explore new hypotheses, and has a chance to go through restructuring. In evolution mode (1) better candidate solutions get stored in more and more attractor networks by cross-network learning and (2) novel candidate solutions are introduced by mutations. The probability of switching between selection and evolution increases with time, but decreases with the number of repeated candidate solutions because of a self-induced mental set.

In this paper, we applied this cognitive architecture to an insight task, the four-tree problem. Experiment 1 served as a benchmark to test our model against human data from Kershaw et al.'s experiment (Kershaw et al., 2013). The model performed similarly to human participants, i.e., there were more solvers in the combined group, which was pre-trained and primed with tetrahedrons than in the control group, which did not receive these treatments. In Experiment 2, three-dimensional training and priming were supposedly less efficient than in Experiment 1. That is, because they involved three dimensional patterns instead of tetrahedrons *per se*. However, we predicted that training and priming would still have a positive effect on problem solving. This proved to be true, however, combined pretraining and priming with three-dimensional patterns was not as effective as we thought, instead the group that received two-dimensional pretraining and three-dimensional priming performed best. This is a prediction that we plan to test in human experiments. In Experiment 3, we showed that deficits in computational capacity and learning abilities of the model decreased solution rate, as it was expected.

### Limitations and future work

The simplification (plant only the fourth tree) and the representation of the problem (100 neurons code additively each coordinate) might be overly simplistic in this study. We plan to work out a more complex representation, where the model searches for the position of all four trees, and with a more realistic coding. We would like to implement this in embodied robots that could physically solve the problem.

We did not explicitly model time in this model (time steps equalled generations). This makes it impossible to model inactivity, which is an important behavioral correlate of impasse. In fact, we did not model impasse *per se* in these simulations. However, we propose that impasse starts sometime before the switch to evolution mode and ends around representational change, because impasse is the phase of problem solving where unconscious thought processes lead to representational change.

Future work should address sampling of candidate solutions to represent solution attempts of human participants. The apparent jump between the goodness of solution attempts of human problem solvers right before the solution can be a result of two different processes. One possibility is that hypotheses gradually increase their fitness through time, but a series of solution attempts does not become conscious, so when one emerges into consciousness, there is an apparent discontinuity. Another possibility is that there is a real jump in the fitness of unconscious hypotheses.

In the present paper, a switch from selectionist to evolutionary dynamics leads to representational change. We are aware of other possibilities, however. A prime candidate could be the re-rendering of the associated adaptive landscape (going beyond adding one more dimension), which would correspond to representational change. Analysis of such alternatives is a task for the future. Another limitation is that switching is unidirectional and happens only once. It would be more realistic to implement a mechanism that can switch back and forth between selection mode and evolution mode.

In Experiment 2, we found that the group that was pretrained with two-dimensional patterns and initialized with three-dimensional patterns performed best, which is unexpected. This might be a limitation of the model, or a valid prediction about a behavior that is like beginner's luck. We plan to test human participants in conditions similar to our Experiment 2 to find out.

We think that the realization of evolutionary processes in the human brain is not impossible. We speculate about the possible components of the cognitive architecture elsewhere (Szilágyi et al., 2016). Here, we would just like to point out that it should be different from Neural Darwinism as it was proposed by Edelman (1987), because he only proposes selection on pre-existing variants, which is a mere one-shot game.

This study shows how semi-neurally implemented evolutionary processes can solve the four-tree problem, and that manipulations lead to increased solution rates just like in human problem solvers. We have some interesting predictions about human behavior, which we will test later. We would also like to implement a more realistic version of the four-tree problem, as well as implementing other insight problems. Our investigations so far show that Darwinian Neurodynamics and its implementation in our cognitive architecture is a promising model for human problem solving.

## Author contributions

AF was responsible for implementing, running and analyzing the experiments and writing the manuscript. IZ and ASz were responsible for implementing the model and writing the manuscript. HV and MÖ were responsible for writing the manuscript. ESz was responsible for the conceptual definition of the model, providing guidance during the implementation of the model and the experiments, writing the manuscript and supervising the work.

### Conflict of interest statement

The authors declare that the research was conducted in the absence of any commercial or financial relationships that could be construed as a potential conflict of interest.
